# Shoulder MRI for Forensic Age Estimation: Ossification Staging of the Proximal Humeral Epiphysis

**DOI:** 10.3390/diagnostics16172751

**Published:** 2026-08-27

**Authors:** Naile Esra Saka, Suna Ors, Marine Dario, Eric Baccino, Sophie Colomb, Laurent Martrille, Oguzhan Ekizoglu

**Affiliations:** 1 Department of Forensic Medicine, Faculty of Medicine, Tekirdag Namik Kemal University, 35090 Tekirdag, Türkiye; nesaka@nku.edu.tr; 2 Department of Radiology, Bakırköy Dr. Sadi Konuk Training and Research Hospital, Health Sciences University, 34147 Istanbul, Türkiye; 3 University Hospitals Sussex NHS Foundation Trust, Worthing BN11 2DH, UK; 4 L’Équipe de Droit Pénal et de Sciences Forensiques de Montpellier (EDPFM), University of Montpellier, 34090 Montpellier, France; 5 Department of Legal Medicine, CHU Montpellier, University of Montpellier, 34000 Montpellier, France; 6 ADES, UMR 7268, Aix-Marseille Université, Bloc A, 13344 Marseille, France

**Keywords:** forensic age estimation, proximal humeral epiphysis, magnetic resonance imaging, skeletal maturation, minimum age

## Abstract

**Background/Objectives:** Magnetic resonance imaging (MRI) of the proximal humeral epiphysis offers a radiation-free alternative for forensic age estimation in living individuals. This study aimed to characterize the relationship between chronological age and epiphyseal maturation using a six-stage ossification classification adapted from the Schmeling–Kellinghaus framework, incorporating one original morphological criterion to distinguish the pre-terminal from the terminal stage of maturation, to establish stage-specific minimum age thresholds, and to evaluate observer reproducibility in a Turkish cohort. **Methods:** In this retrospective, single-center study, 395 shoulder MRI examinations (222 males, 173 females; age range, 12.17–30.67 years) obtained at Bakırköy Dr. Sadi Konuk Training and Research Hospital were evaluated. The proximal humeral epiphysis was staged on T1-weighted turbo spin echo sequences in the coronal oblique plane using a six-stage classification adapted from Schmeling et al. Two radiologists independently assessed all images; intra- and interobserver reliability were determined using Cohen’s kappa. **Results:** Spearman’s rank correlation showed a strong positive association between chronological age and ossification stage (rho = 0.825, *p* < 0.001; males, rho = 0.837; females, rho = 0.808). No significant sex differences in age were found at any stage (*p* > 0.05). The minimum age at Stage 6, the most mature stage observed, was 21.58 years in males and 21.25 years in females. Intra- and interobserver agreement were κ = 0.827 (95% CI, 0.78–0.87) and κ = 0.811 (95% CI, 0.76–0.86), respectively, corresponding to a high level of agreement. **Conclusions:** These findings support MRI staging of the proximal humeral epiphysis as a reliable, radiation-free adjunct for forensic age estimation, with stage-specific minimum ages providing population-specific reference values for application under the minimum age principle. Given the retrospective, single-center, single-scanner design, these results should be regarded as hypothesis-generating data pending prospective, multicenter validation.

## 1. Introduction

Estimating the chronological age of living individuals who lack reliable identity documentation is a recurring challenge in forensic practice. Because skeletal tissues follow a predictable maturation sequence, radiological assessment of specific ossification centers can be used to estimate biological age when documentary evidence is unavailable [1]. Such assessments are required in a variety of legal contexts, including criminal proceedings, asylum applications, adoption, marriage eligibility, and participation in age-restricted competitive sports [2,3,4]. Since many legal systems define criminal responsibility and legal capacity according to specific age thresholds, most commonly 12, 14, 16, 18, and 21 years, accurate age estimation has considerable medico-legal importance [5,6].

Current forensic age estimation protocols for living individuals, including those recommended by the Study Group on Forensic Age Diagnostics (AGFAD), combine physical examination, radiographic assessment of the hand and wrist, dental evaluation, and, when hand skeletal maturation is complete, assessment of the medial clavicular epiphysis using radiography or computed tomography [2,5,7]. However, the use of ionizing radiation solely for administrative or judicial purposes has raised increasing ethical concerns, particularly in children and adolescents [1,3]. Consequently, magnetic resonance imaging (MRI) has emerged as an attractive radiation-free alternative because of its excellent soft-tissue contrast and its ability to directly visualize cartilaginous structures that cannot be adequately assessed using conventional radiography. During the past decade, MRI-based staging systems have been proposed for several anatomical regions, including the knee and the proximal humerus [8,9,10,11,12,13,14].

Compared with these anatomical sites, the proximal humeral epiphysis has received relatively limited attention. Early anthropological investigations based on skeletal collections reported complete epiphyseal fusion at approximately 17–19 years in males and around 17 years in females [15,16,17,18]. The first MRI study specifically evaluating proximal humeral maturation, conducted by Kwong et al., described developmental changes in the humeral growth plate but was limited by a small sample size, a restricted age range, and the absence of a standardized staging system [19]. Subsequently, Zember et al. published a broader review of radiological features and imaging pitfalls in pediatric shoulder maturation, synthesizing evidence from multiple modalities and prior studies rather than presenting a dedicated, primary MRI staging study with its own cohort [20]. More recently, larger MRI studies have applied the Dedouit five-stage classification [21] and the combined Schmeling–Kellinghaus staging system [22] to Turkish populations [8,9], demonstrating good-to-excellent observer agreement while providing preliminary minimum-age estimates for the proximal humeral epiphysis [8,9].

Subsequent investigations have further expanded the available evidence. Vieth et al. proposed a five-stage MRI classification for forensic age assessment of the knee [10], which Altinsoy and Gurses were the first to adapt to the proximal humerus using 3.0-T MRI in a Turkish cohort [11]. Lu et al. later evaluated a large Chinese Han population using a six-stage MRI classification and compared two imaging sequences while developing regression- and classification-based models for discrimination of the 18-year age threshold [12]. Cekdemir et al. subsequently assessed proximal humeral maturation using the Kellinghaus substaging system in another Turkish cohort examined with 1.5-T MRI [13]. More recently, Guo et al. demonstrated the reproducibility of the Vieth classification in a large northern Chinese knee MRI cohort, supporting the robustness of this staging approach across diverse populations [14].

Despite these advances, direct comparison between published studies remains challenging because differences in staging systems, MRI sequences, imaging planes, and study populations may each influence the reported age thresholds [9,10,11,12,13,14,23]. Furthermore, the effects of sex, ethnicity, and socioeconomic background on proximal humeral maturation have not yet been fully clarified [23].

Building upon the methodological framework established by previous MRI studies from our institution [8,9], the present study applies a six-stage ossification classification, adapted from the Schmeling–Kellinghaus staging framework [2,22] and incorporating one original morphological criterion to distinguish the pre-terminal from the terminal stage of maturation (Section 2.3), to a large, sex-balanced cohort of shoulder MRI examinations. The aims were to characterize the relationship between chronological age and proximal humeral epiphyseal maturation, establish minimum age thresholds for each developmental stage, and evaluate the reproducibility of the staging system—including the reproducibility of the newly introduced Stage 5/Stage 6 distinction specifically—by assessing intra- and interobserver agreement in the full available cohort rather than in a preliminary subset. Given the retrospective, single-center, single-scanner design, these findings are intended as hypothesis-generating, population-specific reference values applicable under the forensic minimum age principle—rather than as a general predictive model of chronological age or a substitute for external, multicentre validation.

## 2. Materials and Methods

### 2.1. Study Population

**Figure 1:**
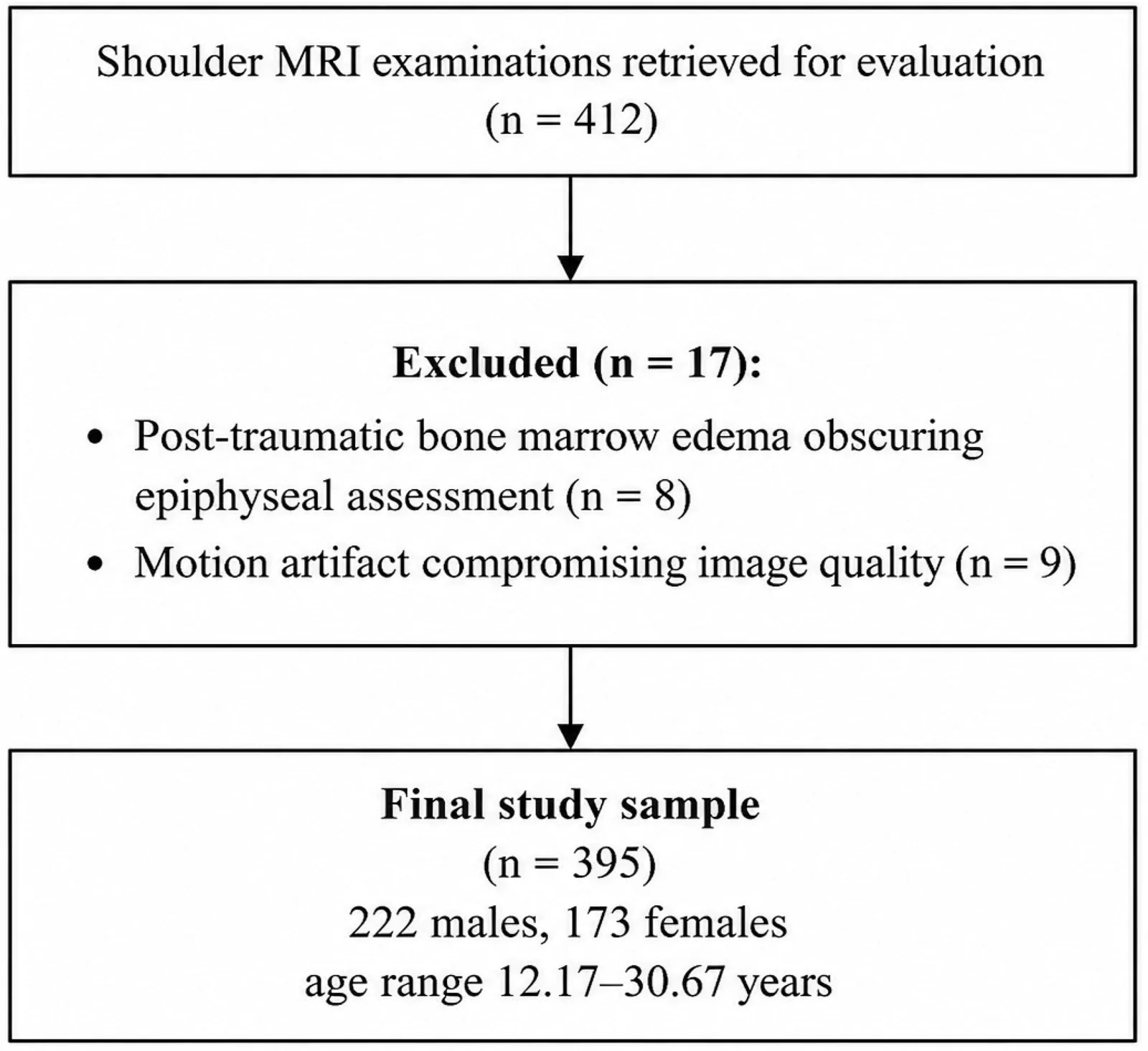
Study workflow showing patient identification, exclusions, and final sample composition.

This retrospective cross-sectional study was conducted at the Department of Radiology, Bakırköy Dr. Sadi Konuk Training and Research Hospital, Istanbul, Turkey, between 1 January 2014, and 1 January 2016. Shoulder MRI examinations of patients aged 12–30 years, referred to the radiology department for evaluation of traumatic or ligamentous shoulder complaints, were retrospectively reviewed. A more granular breakdown of specific clinical indications and pathologies (e.g., rotator cuff pathology, labral injury, instability) was not systematically coded in the radiology archive and could not be retrieved for this cohort. All consecutively available examinations meeting these criteria within the study period were included; no a priori sample-size or power calculation was performed. Of 412 patients initially identified, 8 were excluded due to post-traumatic bone marrow edema obscuring epiphyseal assessment, and 9 were excluded due to motion artifact compromising image quality, yielding a final study sample of 395 individuals (222 males, 173 females) aged 12.17–30.67 years. All patient data were anonymized prior to analysis in accordance with institutional ethical standards. The study workflow is summarized in Figure 1.

The present sample was drawn from the same institutional MRI archive and study period as the authors’ previous reports on the proximal humeral epiphysis (Ekizoglu et al. [8,9]). The present evaluation is nevertheless independent: a different staging system was applied—the six-stage classification described in Section 2.3, distinct from the previously published Dedouit- and Schmeling–Kellinghaus-based analyses—and image evaluation was performed by different observers (Section 2.4). Because each staging system differs in its sensitivity to bone marrow edema and motion artifact, the initial sample and exclusion counts reported here are not directly comparable to those reported previously. Relative to the prior analyses, the present six-stage system incorporates an additional, original morphological criterion refining the distinction between the pre-terminal and terminal stages of maturation (Section 2.3), extending discrimination into older adolescent and young-adult ages.

The left shoulder was evaluated in all cases, consistent with the authors’ previous studies.

Chronological age at the time of MRI was calculated as a decimal value from the interval between the documented date of birth and the date of the MRI examination. Exclusion criteria comprised post-traumatic bone marrow edema and motion artifact compromising image quality. Additional potential confounders of skeletal maturation—including previous shoulder surgery or fracture, tumors, infections, developmental or skeletal abnormalities, endocrine or metabolic disorders, and relevant medication use—were also assessed; none were identified among the included cases.

The sample is described as a Turkish cohort on the basis of recruitment at a single hospital in Türkiye; nationality or ethnicity was not independently verified beyond this recruitment setting.

### 2.2. Image Acquisition

All examinations were performed on a 1.5 T whole-body MRI scanner (Avanto; Siemens, Erlangen, Germany) using a dedicated extremity coil. The proximal humeral epiphysis was assessed on T1-weighted turbo spin echo (T1 TSE) sequences acquired in the coronal oblique plane (TR 500 ms; TE 15 ms; field of view, 150 mm; slice thickness, 2–4 mm; voxel size, 0.5 × 0.5 × 3.5 mm; acquisition time, 1 min 44 s). All available slices were reviewed for each case to allow staging based on the most advanced degree of ossification observed.

### 2.3. Ossification Staging

**Figure 2:**
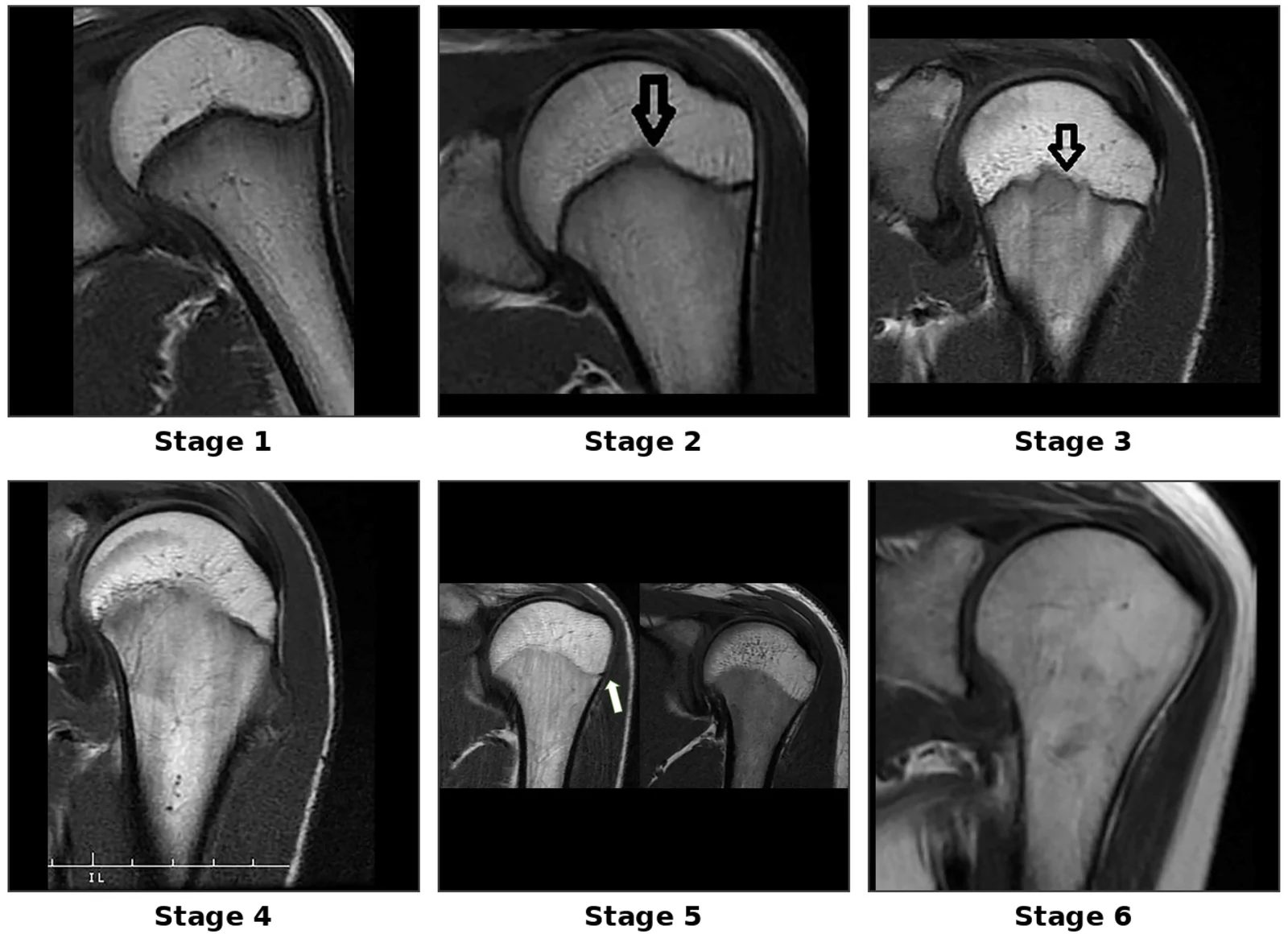
Six ossification stages of the proximal humeral epiphysis on coronal oblique T1-weighted MRI.

The maturation status of the proximal humeral epiphysis was classified using a six-stage system adapted from the staging criteria described by Schmeling et al. [2] and the amplified sub-staging approach of Kellinghaus et al. [22], applied to all 395 included examinations (Figure 2). Stages were defined as follows:

- Stage 1: A continuous intermediate signal band on T1-weighted images with no evidence of osseous bridging between the two structures.
- Stage 2: Epiphyseal–metaphyseal fusion completes one third or less of the former gap between epiphysis and metaphysis.
- Stage 3: Epiphyseal–metaphyseal fusion completes between one third and two thirds of the former gap between epiphysis and metaphysis.
- Stage 4: Epiphyseal–metaphyseal fusion completes over two thirds of the former gap between epiphysis and metaphysis.
- Stage 5: The epiphyseal cartilage is fully ossified, and the epiphyseal scar is dominantly visible, or minimal residual physis remains.
- Stage 6: The epiphyseal–metaphyseal gap is completely bridged by bone, with full disappearance of the cartilaginous growth plate.

Representative images for each stage are shown in the composite comparison in Figure 2.

The six-stage system is based on the previously adapted Schmeling–Kellinghaus staging framework [2,22], to which one original criterion was added: a notch-shaped area of incomplete fusion at the epiphyseal–metaphyseal junction, immediately preceding complete maturation, was incorporated as an independent morphological marker distinguishing the pre-terminal stage (Stage 5) from complete fusion (Stage 6). This addition is original to the present study and extends the system’s discriminatory range into older adolescent and young-adult ages. Stages 1 through 4 of the present system correspond directly, without modification, to Stages 1 through 4 of the Schmeling–Kellinghaus framework [2,22]. The classic Schmeling–Kellinghaus Stage 5 (complete epiphyseal fusion) was subdivided into two stages in the present system—Stage 5 and Stage 6—using the notch-shaped fusion criterion described above to distinguish cases with a residual, incompletely bridged notch (Stage 5) from cases showing complete osseous bridging with no residual growth plate (Stage 6).

### 2.4. Observer Assessment and Reliability

Image evaluation was performed independently by two radiologists (R1, Suna Ors, and R2, Oguzhan Ekizoglu). R1 had 20 years of professional radiological experience, including prior experience in forensic age estimation of living individuals using CT and MRI; R2 had 6 years of experience. To assess reproducibility, all 395 examinations were re-evaluated: interobserver agreement was assessed by comparing the independent scores of R1 and R2, who were blinded to patient identity and to each other’s prior scoring, and intraobserver agreement was assessed by R1 re-scoring the complete sample on a second occasion. Intra- and interobserver agreement were quantified using Cohen’s kappa (κ) statistic. Agreement was interpreted according to the classification proposed by Altman [24]: κ < 0.20, poor; κ = 0.21–0.40, fair; κ = 0.41–0.60, moderate; κ = 0.61–0.80, good; κ = 0.81–1.00, very good.

### 2.5. Statistical Analysis

Statistical analyses were performed using SPSS version 17 software (SPSS, Chicago, IL, USA). Descriptive statistics are presented as mean ± standard deviation, median, minimum–maximum, and lower/upper quartiles, as appropriate. The association between chronological age and ossification stage was assessed using Spearman’s rank correlation coefficient, calculated separately for the total sample and for each sex. Between-sex differences in age at each stage were evaluated using the Mann–Whitney U test. A *p*-value < 0.05 was considered statistically significant.

### 2.6. Ethical Approval

All procedures involving human participants were conducted in accordance with the ethical standards of the institutional research committee and the 1964 Helsinki Declaration and its later amendments. This study was approved by the Bakırköy Dr. Sadi Konuk Training and Research Hospital Clinical Research Ethics Committee (approval no. 2013/123). As a retrospective study using anonymized data, formal individual informed consent was not required.

## 3. Results

### 3.1. Sample Characteristics

The final sample comprised 395 individuals (222 males, 173 females) aged 12.17–30.67 years. Mean age was 22.62 ± 4.05 years in males and 22.66 ± 4.51 years in females, a difference of 0.04 years between sexes. Age distribution by sex, in single-year bands, is presented in Table 1: individuals were distributed across the 12–30-year range, with the largest single-year groups falling between 20 and 25 years in both sexes.

### 3.2. Observer Agreement

Intraobserver agreement for staging of the proximal humeral epiphysis was κ = 0.827 (95% CI, 0.78–0.87), and interobserver agreement was κ = 0.811 (95% CI, 0.76–0.86). According to the classification of Altman [24], these values correspond to very good agreement, indicating that the classification system was applied with high repeatability and consistency by both observers.

### 3.3. Age and Ossification Stage

Stage 1 was observed in 9 individuals (5 males, 4 females); Stage 2 was observed in 6 individuals (3 males, 3 females); Stage 3 was observed in 10 individuals (4 males, 6 females); Stage 4 was observed in 105 individuals (62 males, 43 females); Stage 5 was observed in 106 individuals (67 males, 39 females); Stage 6 was observed in 159 individuals (81 males, 78 females). The proportion of individuals classified as Stage 6 (the most mature stage in this system) increased markedly with age, and this stage accounted for the largest share of the sample overall (159 of 395 individuals, 40.3%), consistent with the predominantly post-pubertal age range of the sample.

Mean age rose progressively across stages in both sexes: from 13.67 years (males)/12.81 years (females) at Stage 1 to 26.58 years (males)/26.14 years (females) at Stage 6. Full descriptive statistics (N, maximum, mean ± SD, quartiles, and median age) for every stage and sex are presented in Table 2 (the minimum-age column has been moved to Table 4 to avoid redundancy).

Spearman’s rank correlation showed a strong, statistically significant positive association between chronological age and stage. Correlation coefficients were rho = 0.825 overall (*p* < 0.001, N = 395), rho = 0.837 in males (*p* < 0.001, N = 222), and rho = 0.808 in females (*p* < 0.001, N = 173).

### 3.4. Sex Differences

Mann–Whitney U comparisons of age between sexes were performed at each stage: Stage 1 (U = 16.0, *p* = 0.190); Stage 2 (U = 5.0, *p* = 1.000); Stage 3 (U = 20.0, *p* = 0.108); Stage 4 (U = 1381.0, *p* = 0.757); Stage 5 (U = 1337.0, *p* = 0.844); Stage 6 (U = 3413.0, *p* = 0.382). No statistically significant sex difference was found at any stage (*p* > 0.05 for all comparisons; Table 3). Sample sizes at Stage 1, Stage 2, and Stage 3 were small (N ≤ 4 in at least one sex), so these specific comparisons should be interpreted with caution.

### 3.5. Minimum Age Thresholds

Minimum observed age increased monotonically with stage in both sexes (Table 4). The youngest age recorded at Stage 6—the most mature stage observed in this sample and the threshold of principal relevance under the forensic minimum age principle—was 21.25 years in females and 21.58 years in males.

**Table 4:** Minimum age thresholds by stage and sex.

Stage	Male (Min Age, Years)	Female (Min Age, Years)
1	12.42	12.17
2	14.25	12.42
3	15.17	14.25
4	15.75	14.58
5	18.25	16.17
6	21.58	21.25

## 4. Discussion

In the present single-center study, MRI assessment of the proximal humeral epiphysis using a six-stage ossification classification demonstrated a strong positive association between epiphyseal maturation and chronological age (rho = 0.825, *p* < 0.001) in a relatively large and sex-balanced Turkish cohort. These findings are consistent with previous studies indicating that the proximal humeral epiphysis is a reliable skeletal indicator for forensic age estimation in living individuals [8,9,11,12,13]. As expected, the mean chronological age increased progressively with advancing maturation stage, supporting the biological validity of the applied staging system. The predominance of individuals in the most mature stage (Stage 6) most likely reflects the retrospective nature of the study, in which shoulder MRI examinations were obtained for clinical indications and therefore predominantly involved post-pubertal individuals, a characteristic similarly reported in previous retrospective MRI investigations [8,9,11].

No statistically significant sex differences were observed at any maturation stage. This finding agrees with previous studies performed using the combined Schmeling–Kellinghaus classification [9] and the recently adapted Vieth classification for the proximal humerus [11], both of which also reported comparable maturation patterns between males and females. In contrast, the earlier study employing the Dedouit classification demonstrated significant sex-related differences in several developmental stages [8]. These discrepancies may be explained by methodological differences, including the staging system, MRI sequence, and stage definitions, all of which may influence the identification of transitional ossification stages [12]. It should also be acknowledged that the limited number of individuals in the earliest maturation stages in the present study reduced the statistical power of sex-specific comparisons within these groups. Therefore, the absence of statistically significant differences in early stages should be interpreted with caution, and confirmation in larger cohorts with a more balanced age distribution would be desirable.

The sex imbalance was not uniform across the age range: males and females numbered 27/27 at 12–17 years, 51/30 at 18–20 years, 83/58 at 21–24 years, and 61/58 at 25–30 years (Table 1), with the imbalance concentrated in the intermediate bands. Notably, the Stage 6 minimum age was slightly lower in females (21.25 years) than in males (21.58 years) despite the smaller female sample, indicating that this pattern is not simply attributable to sample size. Age was retained as a continuous variable in the primary analyses rather than divided into arbitrary bands, since further stratification would leave very few observations per cell—particularly in the already-small Stages 1–3—risking unstable estimates; minimum ages should accordingly be interpreted as sample-specific lower observations rather than fixed biological cut-offs.

Comparison of the minimum age thresholds obtained in the present study with previously published data reveals moderate differences between investigations. However, these differences should not be interpreted as contradictory findings because the available studies differ considerably with respect to staging systems, MRI protocols, image acquisition parameters, study populations, and statistical methodology. Previous Turkish studies applying the Dedouit [8] and Schmeling–Kellinghaus [9] classifications, as well as the more recent adaptation of the Vieth staging system [11], have consistently demonstrated progressive maturation of the proximal humeral epiphysis during late adolescence despite reporting different stage-specific minimum ages. Similarly, Lu et al. [12] reported slightly improved diagnostic performance using T2-weighted fat-suppressed sequences compared with conventional T1-weighted imaging in a Chinese population, whereas Cekdemir et al. [13] observed a predominance of fully mature stages that was largely attributable to the age distribution of their study population. Collectively, these findings suggest that differences in reported age thresholds are more likely related to methodological heterogeneity than to genuine biological disagreement. Accordingly, direct comparison of minimum ages across studies should be performed cautiously, and reference values should always be interpreted within the context of the imaging protocol and population from which they were derived.

From a forensic perspective, particular emphasis should be placed on the minimum age concept rather than on mean chronological ages. Because skeletal maturation demonstrates considerable biological variability, individuals of the same chronological age may present with different maturation stages. Consequently, minimum observed ages provide the most conservative basis for medico-legal interpretation and reduce the risk of overestimating chronological age. The present findings therefore support the use of stage-specific minimum ages as complementary reference values in forensic age estimation. Nevertheless, MRI findings of the proximal humeral epiphysis should not be interpreted in isolation. Consistent with current international recommendations, skeletal MRI should be regarded as one component of a multidisciplinary age assessment that incorporates physical examination, dental evaluation, and, where appropriate, assessment of additional skeletal maturation indicators. Such an integrated approach is particularly important when evaluating legally relevant age thresholds, including the age of majority.

Intra- and interobserver agreement were calculated as κ = 0.827 (95% CI, 0.78–0.87) and κ = 0.811 (95% CI, 0.76–0.86), respectively, corresponding to very good agreement according to the Altman classification [24]. These findings are comparable to those reported in previous Turkish cohorts [8,9,11] and further support the reproducibility of the six-stage classification when applied by trained observers using standardized MRI protocols. High observer agreement is particularly important in forensic practice because reproducibility directly influences the reliability and legal defensibility of expert opinions.

Several limitations should be acknowledged. First, the present sample was drawn from the same institutional archive as the authors’ previous reports on the proximal humeral epiphysis [8,9]; although the present evaluation is independent—employing a different staging system and different observers, as detailed in Section 2.1—readers should be aware of this shared institutional source when comparing findings across these publications. Second, because all examinations were obtained for clinical indications (predominantly traumatic or ligamentous shoulder complaints) rather than from healthy volunteers, the sample is a clinically selected population; the extent to which the underlying complaints or any associated pathology influenced skeletal maturation cannot be fully excluded, which constrains generalizability to the general population. Specific diagnostic and pathology data beyond the broad categories used for exclusion (post-traumatic bone marrow edema, motion artifact) were not systematically coded in the retrospective radiology archive and could therefore not be retrieved for the included cohort; consequently, a quantitative breakdown of the underlying clinical indications (e.g., rotator cuff pathology, labral injury, or instability) cannot be provided, and this should be regarded as a further limitation of the retrospective design. Third, the number of individuals in the earliest maturation stages (Stages 1–3; n = 9, 6, and 10, respectively) was small, which limits the precision of stage-specific estimates—particularly minimum ages—in this range and warrants cautious interpretation. Fourth, while the six-stage classification builds on the previously adapted Schmeling–Kellinghaus framework with one original morphological criterion (Section 2.3), this specific combination has not, to our knowledge, been independently externally validated; the reported thresholds should accordingly be regarded as requiring confirmation. Relatedly, although overall intra- and interobserver agreement was very good (Section 3.2), case-level observer scoring data were not retained in a form permitting a stage-pair-specific reliability analysis; consequently, the reproducibility of the Stage 5/Stage 6 distinction specifically—the boundary at which the original criterion introduced in the present study is applied—could not be isolated from the overall agreement statistic and should be evaluated directly in future studies designed to capture case-level, stage-pair-specific agreement. Fifth, minimum observed ages are, by definition, driven by single cases and are therefore sensitive to sampling variation—a single younger individual in a future cohort could lower a reported threshold—and should be interpreted as descriptive of the present sample rather than as fixed population parameters. Sixth, participants were described as a Turkish cohort on the basis of recruitment at a single institution in Türkiye rather than independently verified nationality or ethnicity data; this label should therefore be understood as a proxy for the recruitment setting. Finally, no independent or external validation cohort was available in this study; the reported associations and thresholds therefore require confirmation in separate populations before broader forensic application.

Taken together, these findings support the forensic relevance of proximal humeral MRI staging, subject to the limitations outlined above; the principal conclusions are summarized in Section 5.

## 5. Conclusions

This study demonstrates that MRI-based staging of the proximal humeral epiphysis, using a six-stage ossification classification, is strongly associated with chronological age and can be applied reproducibly by trained observers. No significant sex-related differences in maturation timing were identified, and minimum age thresholds increased consistently with advancing stage in both sexes. These findings support the use of proximal humeral MRI as a supplementary tool in forensic age estimation of living individuals, particularly when applied according to the minimum age principle rather than as a standalone predictor of chronological age. Given the retrospective design, the single-center origin of the sample, and the absence of data on socioeconomic and nutritional status, the reference values presented here should be regarded as population-specific and hypothesis-generating rather than universally applicable. Prospective, multicentre studies incorporating standardized imaging protocols, external validation of the staging system, and more ethnically diverse populations are needed to confirm these findings and to further define the role of proximal humeral MRI within a multidisciplinary forensic age assessment framework.

## Figures and Tables

**Table 1:** Age distribution by sex. Age is grouped by the completed-year component.

Age (Years)	Male (N)	Female (N)	Total (N)
12	1	4	5
13	2	0	2
14	2	7	9
15	7	1	8
16	7	10	17
17	8	5	13
18	10	9	19
19	16	8	24
20	25	13	38
21	25	24	49
22	18	11	29
23	24	14	38
24	16	9	25
25	17	16	33
26	9	9	18
27	4	7	11
28	10	8	18
29	13	9	22
30	8	9	17

**Table 2:** Descriptive statistics for the proximal humeral epiphysis, by stage and sex. LQ, lower quartile; UQ, upper quartile (minimum-age column removed; see Table 4).

Stage	Sex	N	Max	Mean ± SD	LQ	UQ	Median
1	Male	5	15.33	13.67 ± 1.12	13.08	14.17	13.33
1	Female	4	14.08	12.81 ± 0.87	12.29	13.02	12.5
2	Male	3	15.58	15.00 ± 0.68	14.71	15.38	15.17
2	Female	3	16.17	14.45 ± 1.89	13.59	15.46	14.75
3	Male	4	16.67	15.71 ± 0.67	15.29	15.92	15.5
3	Female	6	16.67	14.96 ± 0.91	14.35	15.11	14.67
4	Male	62	23.58	19.77 ± 2.17	18.1	21.33	20.25
4	Female	43	23.17	19.58 ± 2.11	18.09	21.25	20.08
5	Male	67	30.33	21.88 ± 2.16	20.33	23.21	21.67
5	Female	39	28.58	21.94 ± 3.23	20.04	23.33	21.5
6	Male	81	30.58	26.58 ± 2.34	24.58	28.58	26.33
6	Female	78	30.67	26.14 ± 2.74	24.27	28.52	25.62

**Table 3:** Mann–Whitney U test comparing age between sexes at each stage.

Stage	N (Male)	N (Female)	U	*p*
1	5	4	16	0.19
2	3	3	5	1
3	4	6	20	0.108
4	62	43	1381	0.757
5	67	39	1337	0.844
6	81	78	3413	0.382

## Data Availability

The data presented in this study are available on reasonable request from the corresponding author. The data are not publicly available due to institutional and patient-privacy restrictions on this retrospective clinical imaging dataset.
